# Current Status and Trends of Research on Anthracycline-Induced Cardiotoxicity from 2002 to 2021: A Twenty-Year Bibliometric and Visualization Analysis

**DOI:** 10.1155/2022/6260243

**Published:** 2022-08-11

**Authors:** Yu Wang, Yifei Rao, Zhijian Lin, Rina Sa, Yuling Yin, Xiaomeng Zhang, Bing Zhang

**Affiliations:** ^1^School of Chinese Materia Medica, Beijing University of Chinese Medicine, Beijing 100029, China; ^2^Center for Pharmacovigilance and Rational Use of Chinese Medicine, Beijing University of Chinese Medicine, Beijing 100029, China

## Abstract

Anthracyclines constitute the cornerstone of numerous chemotherapy regimens for various cancers. However, the clinical application of anthracyclines is significantly limited to their dose-dependent cardiotoxicity. A comprehensive understanding of the current status of anthracycline-induced cardiotoxicity is necessary for in-depth research and optimal clinical protocols. Bibliometric analysis is widely applied in depicting development trends and tracking frontiers of a specific field. The present study is aimed at revealing the status and trends of anthracycline-induced cardiotoxicity during the past two decades by employing bibliometric software including R-bibliometric, VOSviewer, and CiteSpace. A total of 3504 publications concerning anthracycline-induced cardiotoxicity from 2002 to 2021 were collected from the Web of Science Core Collection database. Results showed significant growth in annual yields from 90 records in 2002 to 304 papers in 2021. The United States was the most productive country with the strongest collaboration worldwide in the field. Charles University in the Czech Republic was the institution that contributed the most papers, while 7 of the top 10 productive institutions were from the United States. The United States Department of Health and Human Services and the National Institutes of Health are the two agencies that provide financial support for more than 50% of sponsored publications. The research categories of included publications mainly belong to Oncology and Cardiac Cardiovascular Systems. The Journal of Clinical Oncology had a comprehensive impact on this research field with the highest IF value and many publications. Simunek Tomas from Charles University contributed the most publications, while Lipshultz Steven E. from the State University of New York possessed the highest *H*-index. In addition, the future research frontiers of anthracycline-induced cardiotoxicity might include early detection, pharmacogenomics, molecular mechanism, and cardiooncology. The present bibliometric analysis may provide a valuable reference for researchers and practitioners in future research directions.

## 1. Introduction

Anthracyclines (ANTs) are recognized as the most effective antineoplastic antibiotics. They have constituted the backbone of regimens for numerous solid and hematological tumors since the 1960s, including ovarian, breast cancer, lymphomas, sarcoma, and pediatric leukemia [1]. Doxorubicin (DOX) is the representative drug of ANTs, which account for nearly 60% of pediatric cancer regimens and contribute to the overall 5-year average survival in over 80% of patients [2, 3]. Unluckily, severe side effects were recognized immediately after using ANTs in clinical practice. The clinical use of ANTs is greatly limited to their multiorgan toxicities, mainly including cardiotoxicity, liver injury, pulmonary lesion, testicular toxicity, and brain damage [4]. Among those adverse reactions, the time- and dose-dependent cardiotoxicity is the most serious [5]. As reported, cumulative DOX doses of 400, 550, and 700 mg/m^2^ could increase the incidence of congestive heart failure by 4.7%, 26%, and 48% [6] according to cardiotoxicity which represents a severe adverse effect that may be lethal.

Even so, anthracycline-induced cardiotoxicity (ACT) is still a broad term without an exact definition. It may encompass any cardiac complications caused by ANTs, containing left ventricular dysfunction, congestive heart failure, and irreversible cardiomyopathy [7]. Unfortunately, available therapies against ACT only consist of cardioprotective agents and symptomatic treatments. They are far insufficient in reducing ACT. The significance of ACT treatment raises much attention, and researchers have made many efforts. One of the most representative is the constant introduction of new concepts and techniques in ACT fields, such as cardiooncology, pharmacogenomics, and cardiac gene therapy. Among them, cardiooncology is proposed to achieve a balance between cancer therapy and ACT prevention [8]. Meanwhile, considering the individual differences, pharmacogenomics, a high-throughput microarray technology, has rapidly been applied in the ACT to optimize individualized clinical protocols by elucidating disparities in genetics [9]. In addition, different from traditional therapies, cardiac gene therapy is a profitable attempt to confer long-term cardiac protection by rendering the heart resistant to ANTs [10]. However, most of these studies are in an initial stage and deserve more concern. Thus, a comprehensive understanding of the current status of ACT is necessary for in-depth research and optimal clinical protocols.

Bibliometric analysis has emerged as the method to depict the knowledge structures and developmental trends of a specific field based on statistical and visualization techniques [11, 12]. It could quantitatively identify research trends and hot topics from publication information, estimate the productivity of authors and institutions, and determine international cooperation and geographic distributions [13, 14]. Nowadays, thousands of papers concerning ACT have been published, indicating researchers' sustained interest in this field. However, a bibliometric analysis of ACT is yet to be seen. Therefore, the present study performed a bibliometric analysis of ACT during the past two decades. We hope this study may reveal the status and trends of ACT and provide valuable references for further research.

## 2. Material and Methods

A bibliometric study usually contains two steps, data collection, and bibliometric and visualized analysis [15–17]. For data collection, the Web of Science Core Collection (WoSCC) database (http://apps.webofknowledge.com), one of the most influential databases of scientific publications, is commonly considered in bibliometric analysis for its comprehensive information [18, 19]. For bibliometric and visualized analysis, CiteSpace, VOSviewer, and *R*-bibliometrix are the most frequently used software [20–22]. In this step, the critical information of included publications would be extracted, which usually contains countries/regions, institutions, authors, journals, keywords, and cocited references [23]. From cooperative networks, the most contributed authors, institutions, and countries/regions would be identified, and the interested journals also could be screened out. These results would point out several prominent research groups in a specific field and benefit from tracking their studies for an in-depth exploration. Keywords and cocited references are usually the focus of bibliometric analysis. Their co-occurrence networks and burst terms could reflect the developmental process and hot frontiers of a specific research field [24, 25].

### 2.1. Search Strategy and Data Collection

**Figure 1:**
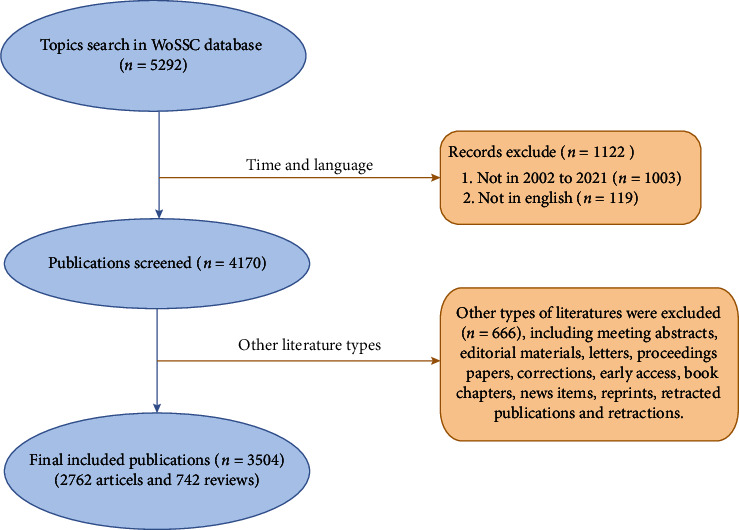
Flowchart of data filtration processing and excluding publications.

The related publications were obtained from the Science Citation Index Expanded (SCI-E) through WoSCC on May 11, 2022. The search terms were determined following synonyms from Medical Subject Headings (MESH, https://www.ncbi.nlm.nih.gov/mesh) and text words. In the present study, the retrieval strategy was as follows: Topics = “cardiotoxicit^∗^,” “cardiac toxicit^∗^,” “toxicit^∗^, cardiac,” “cardiac damage^∗^,” “cardiac injur^∗^,” “heart damage^∗^,” “heart injur^∗^,” “arrhythmias,” “angina pectoris,” “myocardial ischemia,” “acute myocardial infarction,” “sudden death,” “heart failure,” “left ventricular dysfunction,” “chronic cardiac failure,” and “anthracycline^∗^.” Articles and reviews published from 2002 to 2021 in English written were considered. The particular retrieval procedure is illustrated in Figure 1. Ultimately, a total of 3504 publications were included to further analysis.

### 2.2. Bibliometric and Visualized Analysis

CiteSpace, VOSviewer, and *R*-bibliometrix were applied for literature analysis and bibliometric visualization in the present study. Microsoft Excel (Office 2019) was employed to manage data and draw figures after data deduplication with CiteSpace.

CiteSpace (version 5.8.R3) is a free software for visualizing networks of document citations, collaboration relationships, and research hotspots [26, 27]. We performed the burst analysis of keywords and the cocitation analysis of references with CiteSpace. The parameters of CiteSpace were as follows: link retaining factor (LRF = 5), look back years (LBY = 10), *e* for top *N* (*e* = 2), years per slice (1), selection criteria (*g*-index, *k* = 25), and pruning (pathfinder + pruning sliced network + pruning the merged network). VOSviewer (version 1.6.17) is another commonly used bibliometric tool that analyzes the collaborative relationships of countries/regions, institutions, and authors [28]. We employed VOSviewer to construct the coauthorship networks of countries/regions, institutions, authors, and the co-occurrence network of keywords. The parameters of VOSviewer were as follows: the counting method was full counting, and the threshold (*T*) depended on corresponding observed subjects. *R*-bibliometrix (version R 4.1.2) can be used for quantitative analyses in the map of country collaboration, trend topics, and thematic map of keywords [29]. In addition, we used the *H*-index to quantify the scientific output and influence of researchers [30, 31]. The journal impact factors (IF), a marker for evaluating journals' influence, were obtained from the 2020 Journal Citation Reports (JCR) [32].

The present study is a bibliometric analysis of existing publications and does not require ethical approval.

## 3. Results

### 3.1. Annual Output of Publication

**Figure 2:**
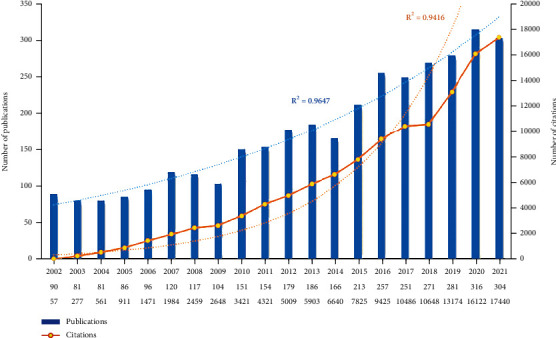
Annual number of publications and citations. Each bar in blue represents the number of publications per year. Each node in yellow means the number of citations per year. Dotted lines in blue and orange, respectively, represent the growth curve of publications and citations.

A total of 3504 publications were obtained, including 2762 articles and 742 reviews. As shown in Figure 2, the annual yields grew more than three times in the past two decades, from 90 records in 2002 to 304 papers in 2021. Based on the WoSCC database, the 3504 publications were cited 120782 times, and each article has been cited an average of 34.47 times. The time curve constructed by the logistic regression model suggested a steady growth output, implying researchers' strong interests in the ACT field.

### 3.2. Distribution of Countries/Regions and Academic Collaboration

**Figure 3:**
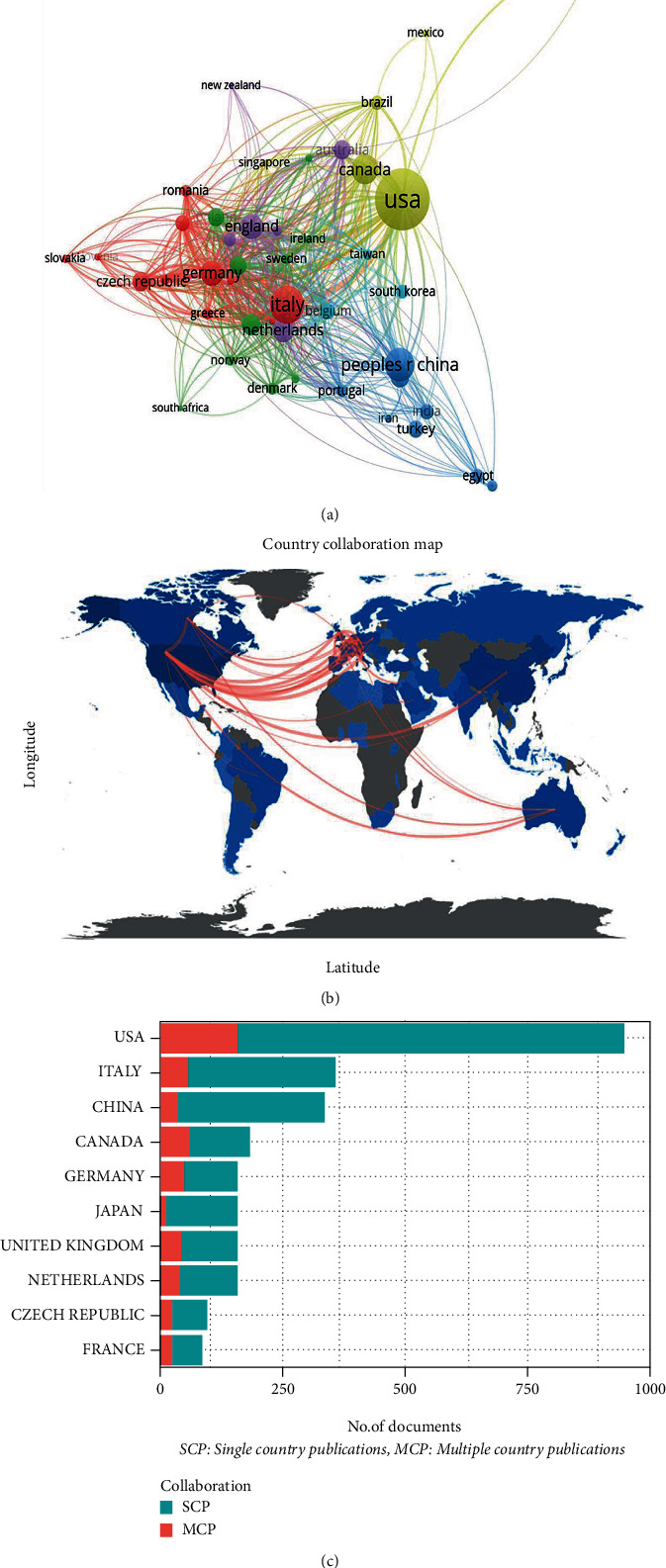
Coauthorship analysis of countries/regions. (a) Overlay map of countries/regions with more than 10 publications. Each node represents a country/region. The size of each node is proportional to the total number of publications. The same color of clusters represents more active cooperation. Lines between two nodes represent the cooperation between two countries/regions. (b) Geographical map in production and collaboration of countries/regions. Different shades of blue indicate different productivity rates, and the darker the blue represents more publications, while grey means no publications. Red lines represent cooperating relations between countries/regions, and the thicker line means stronger cooperation. (c) Histogram of collaboration status in the top 10 productive countries/regions. The *x*-axis represents the number of publications. The *y*-axis represents different countries/regions. Publications cooperated with multiple countries/regions (MCP) are plotted by the red part, while the green part means publications with a single country/region (SCP).

Researchers from 82 countries/regions published 3504 publications. Half of the top 10 productive countries/regions are in Europe, 1 in Oceania, and the other 4 in North America and Asia. As shown in Table 1, the United States contributed the greatest number of publications (*n* = 1154, 32.93%), followed by Italy (*n* = 442, 12.61%) and China (*n* = 343, 9.79%), and they accounted for more than half of the total publications in the field of ACT during 2002 to 2021. Besides, publications from the United States owned the highest citations (*n* = 55528), followed by those from Italy (*n* = 23065) and Canada (*n* = 16395). Forty-four countries/regions with the threshold of ten documents in the coauthorship network were displayed in Figure 3(a). The size of nodes was consistent with the number of publications. In addition, Figure 3(b) visualizes the world map of publications in ACT research. The symbol that darker the blue represents the more publications, and the thicker the red line means the stronger cooperation between countries/regions. It illustrated an active and strong collaboration among different countries/regions. More detailed information in Figure 3(c) suggests that the United States, Italy, and Canada were the top 3 countries with the strongest cooperation during the survey period.

### 3.3. Contribution of Institutions and Academic Collaboration

**Figure 4:**
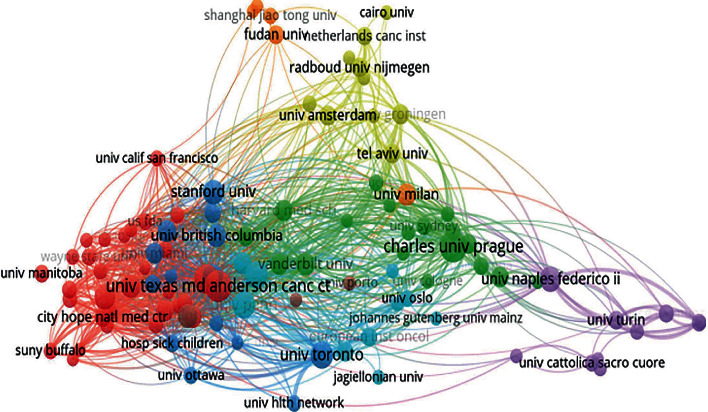
Overlay map of institutions with more than 15 publications. Each node represents an institution. The size of each node is proportional to the occurrence frequency of institutions among included publications. The same color of clusters represents more active cooperation. Lines between two nodes represent the cooperation between two institutions.

A total of 3806 institutions were involved in the present study. Seven of the top 10 institutions came from the United States, and the remaining were from the Czech Republic, Canada, and Italy, respectively. As shown in Table 2, Charles University (*n* = 74, 2.11%) contributed the most papers, followed by the University of Texas MD Anderson Cancer Center (*n* = 73, 2.08%) and Memorial Sloan Kettering Cancer Center (*n* = 63, 1.80%). The coauthorship network consisting of 101 institutions was visualized with the threshold of fifteen records and displayed in Figure 4. Institutions symboled with the same color had more active cooperation during the survey.

### 3.4. Contribution of Authors

**Figure 5:**
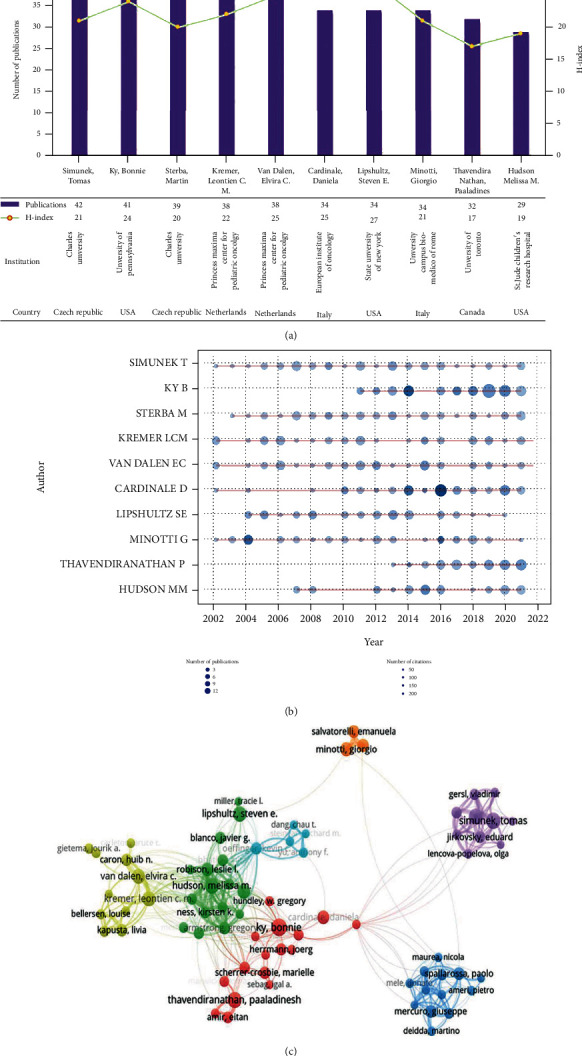
Coauthorship analysis of authors. (a) The top 10 productive authors and their *H*-index. Each bar in purple represents the number of publications of each author. Each node in yellow means the *H*-index of each author. (b) The annual production number of the top 10 productive authors. The *x*-axis represents years. The *y*-axis represents different authors. Each node represents the productivity of each author per year. The bigger the size and the darker the color of each node are, respectively, proportional to the number of publications and citations. (c) Overlay map of authors with over 10 publications. Each node represents an author. The size of each node is proportional to the productivity of authors. The same color of clusters represents more active cooperation. Lines between two nodes represent the cooperation between two authors.

A total of 17422 researchers contributed to the 3504 publications in the ACT field from 2002 to 2021.  Figure 5(a) exhibits the number of publications of the top 10 productive authors and their *H*-index. Simunek Tomas from Charles University contributed the most papers (*n* = 42), followed by Ky Bonnie from the University of Pennsylvania (*n* = 41) and Sterba Martin from Charles University (*n* = 39), while Lipshultz Steven E. from the State University of New York owned the highest *H*-index (27), followed by van Dalen Elvira C. from the Princess Máxima Center for Pediatric Oncology (25) and Cardinale Daniela from the European Institute of Oncology (25). More detailed information concerning each author's publications over time is shown in Figure 5(b). The node size indicates the number of publications. And the color of the nodes is light to dark blue, meaning that the number of citations is small to large. The coauthorship network of 82 authors with more than 10 papers was visualized in Figure 5(c), demonstrating widespread cooperation among different groups of ACT research.

By tracking the specific research area of productive authors and scanning their articles, we may quickly gain insight into the ACT field. Simunek Tomas and his colleagues mainly concentrate on the mechanism and discovery of cardioprotective agents against ACT. They clarified the cardioprotective mechanism of dexrazoxane, the only clinically approved drug against ACT, is the interaction with topoisomerase-II*β* (Top2*β*) but not metal chelation and protection against direct oxidative damage [33]. They also found a water-soluble prodrug of dexrazoxane analogs (ICRF-193), named GK-667 for short, which is a promising agent against ACT [34]. Lipshultz Steven E. paid more attention to the clinical observation and management of ACT in children. He and his colleagues advocated using dexrazoxane to limit ACT in children and adolescents with cancer for the opinion of no “safe” dose of ANTs [35].

### 3.5. Analysis of Academic Journals

**Figure 6:**
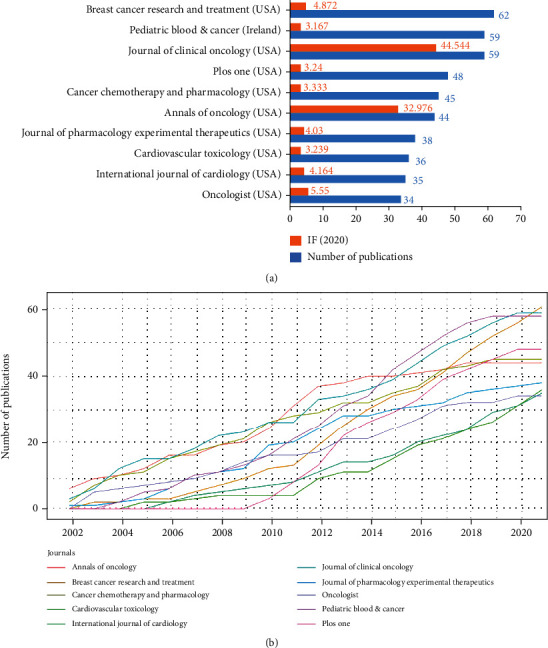
Analysis of journals. (a) The top 10 academic journals and their IF values. The vertical axis represents academic journals. The orange and blue bars indicate the journal's IF2020 value and the number of publications. (b) The annual production of the top 10 academic journals. The *x*-axis represents years. The *y*-axis means the number of publications. Different color lines stand for different academic journals.

The 3504 publications were published in 857 academic journals. The top 10 academic journals published the most papers in the ACT field, and their IF values are displayed in Figure 6(a). Nine of those ten journals were from the United States. Breast Cancer Research and Treatment published the most papers (*n* = 62), followed by Pediatric Blood & Cancer (*n* = 59) and Journal of Clinical Oncology (*n* = 59). And Journal of Clinical Oncology also showed a comprehensive impact on tumor research with the highest IF (IF2020 = 44.544), followed by Annals of Oncology (IF2020 = 32.976) and Oncologist (IF2020 = 5.55).  Figure 6(b) displays an increasing growth of publications concerning ACT research in the top 10 journals over the past decades.

The analysis of academic journals reflected areas that are interested in the ACT. Consistent with the therapeutic area of ANTs, ACT obtained more attention from journals specializing in breast cancer and pediatric blood cancer. In addition, ANTs continually receive attention from the clinical oncology field, and the hotness of ACT has risen in the cardiovascular, chemotherapy, and pharmacology field.

### 3.6. Analysis of Research Categories and Funding Agencies

**Figure 7:**
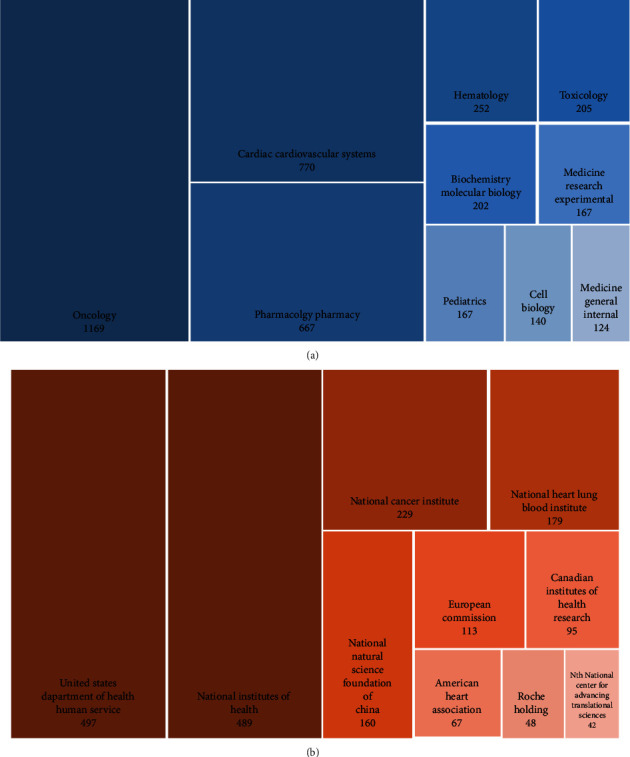
Analysis of research categories and funding agencies. (a) The top 10 research categories. (b) The top 10 funding agencies. Block with darker color represents a greater number of documents.

According to the WoSCC database, 89 categories were involved in this research field. From the treemap of the top 10 research categories in Figure 7(a), oncology owed the most frequent occurrence (*n* = 1169), followed by cardiac cardiovascular systems (*n* = 770) and pharmacology pharmacy (*n* = 667).

1939 of the 3504 publications in the present study obtained funding support. As shown in Figure 7(b), two funding agencies from the United States, the United States Department of Health and Human Services and the National Institutes of Health, provide financial support for more than 50% of those sponsored publications (*n* = 497, 489). The other funding agencies contributed more than 100 documents, including the National Cancer Institute (*n* = 229), National Heart Lung Blood Institute (*n* = 179), National Natural Science Foundation of China (*n* = 160), and European Commission (*n* = 113).

### 3.7. Detection and Analysis of Keywords

**Figure 8:**
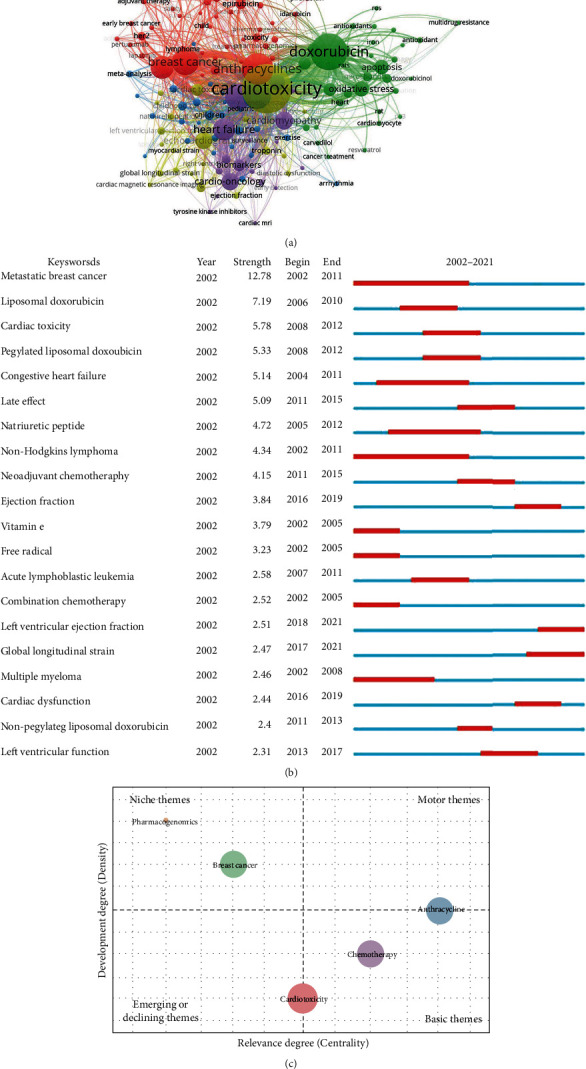
Analysis of keywords. (a) Co-occurrence analysis of keywords with a threshold over 10. Each node represents a keyword. The size of each node is proportional to the occurrence frequency of a keyword. The same color of nodes represents the same cluster. Lines between two nodes represent the relevance of two keywords. (b) Burst analysis of the top 20 keywords. The blue line represents the period from 2002 to 2021, while the red line plots the periods of each burst keyword. (c) Thematic map in the ACT field. The four quadrants of the two-dimensional diagram of which the motor themes (Q1), the highly developed and isolated themes (Q2), the emerging or declining themes (Q3), and the basic and transversal themes (Q4). Each colored bubble represents a cluster of correlative keywords. The bubble size is proportional to the occurrence frequency of associated keywords. The horizontal axis represents the links from one cluster to others, called centrality, and the vertical axis demonstrates the strength of these links, also named density.

Keywords could most accurately reflect the topic of an article and mirror the research frontiers of a research field. A total of 4498 keywords from authors were involved in the present study. VOSviewer generated the co-occurrence analysis network of authors' keywords. As shown in Figure 8(a), there were 154 authors' keywords with a threshold over 10 frequencies. The leading 10 keywords and their frequency of occurrence were as follows: cardiotoxicity (1129), doxorubicin (646), anthracyclines (491), breast cancer (353), chemotherapy (339), trastuzumab (268), heart failure (248), cardiomyopathy (170), cardiooncology (157), and echocardiography (152). The cluster analysis symbolized those keywords into five clusters with different colors. Specifically, terms related to the treatment of ANTs were in red, mainly including breast cancer, acute myeloid leukemia, and lymphoma. The largest node with DOX in green may relate to its frequent occurrence in mechanism studies. Terms associated with clinical studies were in blue, mainly consisting of epidemiology, meta-analysis, and risk factors. Words concerning evaluated ACT indexes were in yellow, mainly comprising left ventricular ejection fraction (LVEF), brain natriuretic peptide (BNP), and global longitudinal strain (GLS). Terms correlated with ACT detection were in purple, mainly composed of cardiomyopathy, biomarkers, and early detection.

The burst analysis refers to detecting keywords with a high frequency of occurrence in a certain period which could reflect the evolution trend of specific research.  Figure 8(b) illustrates the burst analysis of keyword in a three-year slice from 2002 to 2021. The strength of the top 20 keywords with the strongest bursts varied from 2.31 to 12.78. The duration ranged from 3 to 10 years. The term metastatic breast cancer and the word non-Hodgkin's lymphoma occupied a long time from 2002 to 2011 with a burst strength of 12.78 and 4.34, respectively, implying continuous research attention. In recent years, terms of LVEF and GLS which appeared no less than three years ago might be considered essential topics in the research field of ACT.

The thematic map of ACT was further visualized. The keywords' relative locations, characterized by density and centrality, represented the theme's evolution [36]. The upper right quadrant (Q1) reflects motor themes that usually are important and well developed. The upper left quadrant (Q2) displays highly developed themes, albeit with a certain degree of isolation. The lower left quadrant (Q3) shows the emerging or declining themes. The lower right quadrant (Q4) always represents basic or transversal themes. As shown in Figure 8(c), the term ANTs is sandwiched between Q1 and Q4, demonstrating that the topic is well developed and can structure the research field. At the same time, the cardiotoxicity term sandwiched between Q3 and Q4 may indicate that some of its components are basic for developing the research field of ACT. The term chemotherapy in Q3 as the basic topic in ANTs research is unsurprised. Moreover, pharmacogenomics and breast cancer in Q2 imply the highly developed internal bonds but still of marginal contribution to the development of the ACT field. The thematic analysis suggests that more efforts should be made to connect pharmacogenomics and ACT, and breast cancer may be a disease specie that should be focused on. According to reports, understanding the genetic factors predisposing patients to poor treatment outcomes will help guide personalized treatment to obtain maximal benefit [37]. Pharmacogenomics represents a promising area of research in this context [38].

Overall, keyword analysis could obtain the developmental trajectory of ACT, including the main research areas, current research concerns, and future research trends. For instance, we classified keywords of the ACT research into five clusters by sorting their occurrence frequency. Among them, two clusters colored yellow and purple represent the ACT indexes and ACT detections, revealing the two focused areas in the ACT field. Subsequently, the burst analysis discovered the updated changes in detect indicators from left ventricular function into GLS, which indicated the current research concerns of developing novel detect markers. Combined with the result of the thematic map in the ACT field, pharmacogenomics may be an advanced technology that could discover potential early biomarkers in future research.

### 3.8. Analysis of Cocited References

**Figure 9:**
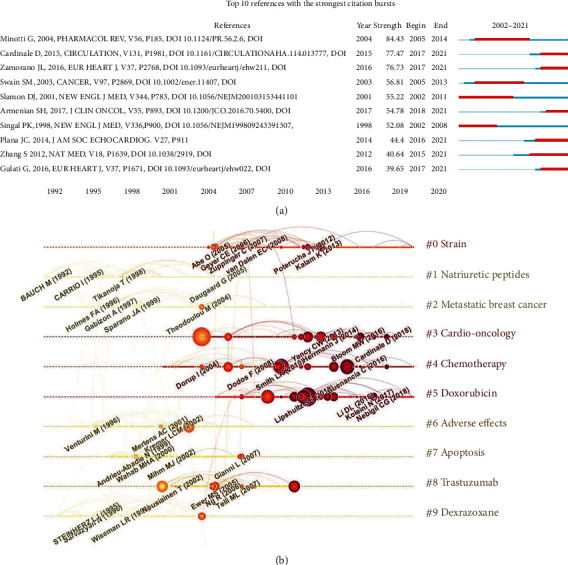
Analysis of cocited references. (a) Burst analysis of top 10 cocited references. The blue line represents the period from 2002 to 2021, while the red line represents the time interval of each burst cocited reference. (b) The visualized timeline view of cocitation clusters in the ACT field. Each horizontal line represents a cluster, and the cluster's label is located at the rightmost end of the line. Each node means a reference, and the larger radius, the more citations. The timeline at the top of the figure and the year corresponding to the node is its publication time. The darker color of the reference's label represents the more recent literature. The link between nodes represents the cocitation relationship.

The burst analysis and cluster analysis of cocited references were conducted with CiteSpace software. The top 10 references with high cocitations are listed in Table 3. Each of them was cocited over 180 times. At the same time, the top 10 cocited references with the strongest citation bursts were exhibited in Figure 9(a). An article by Zhang et al. in Nature medicine in 2012 obtained the most cocitation (*n* = 365) and a high burst (strength = 40.64) with a duration from 2015 to 2021. It identified the cardiomyocyte-specific deletion of Top2*β* could protect cardiomyocytes from DOX-induced DNA double-strand breaks and transcriptome changes. Its high cocitation and burst strength indicate the continuous interest in the ACT mechanism. Similarly, a mechanism review named “anthracyclines: molecular advances and pharmacologic developments in antitumor activity and cardiotoxicity” published by Minotti et al. in Pharmacological Reviews in 2004 got the highest cocitation (strength = 84.43) with a ten-year duration.

The cluster analysis of cocited references may help to understand the common research topics of similar references. And the timeline view of clusters could further reveal research hotspots following a chronological point.  Figure 9(b) displays the 10 essential clusters concerning the research field of ACT based on the loglikelihood ratio algorithm. Each node means a reference, and the larger radius, the more citations. The lines with darker color stand for the latest date. In the present study, modularity *Q* (0.8417 > 0.3) and mean silhouette value (0.9468 > 0.7), two crucial parameters in cluster analysis, represent significance in cluster structure and convincing cluster results. The research hotspots of ACT changed over time. Terms with lighter color symbolize relatively early research hotspots, including natriuretic peptides (#1), metastatic breast cancer (#2), adverse effects (#6), apoptosis (#7), trastuzumab (#8), and dexrazoxane (#9). Terms with darker color may represent recent principal hotspots involving strain (#0), cardiooncology (#3), chemotherapy (#4), and doxorubicin (#5). The detailed cluster information is listed in Table 4.

From the analysis of the cocited references, DOX is still a highly effective and commonly used chemotherapy agent. There was continued interest in ACT mechanisms, early detection, and prevention research. The emerging field of cardiooncology has shown interest in the ACT field and aims to make significant breakthroughs.

## 4. Discussion

### 4.1. The Basic Information of ANTs

ANTs, the archetypal representatives of the tetracyclic type II polyketide natural antibiotics [39–41], were initially isolated from the bacterium Streptomyces peucetius and showed remarkable anticancer activities [42]. The first ANT was named daunorubicin (DNR) and was applied to acute leukemia treatment in 1963 [43]. Subsequently, a precursor substance of DNR, called DOX, was launched with enhanced anticancer activities of leukemia and solid tumors in 1969 [44]. From the perspective of molecular structure, DNR and DOX are both comprised of the aminosugar daunosamine and anthracene nucleus. The only difference is the side chain of DOX terminates with primary alcohol while DNR with methyl, which expands the spectrum of anticancer activities of DOX [45]. The anticancer mechanism of DNR and DOX is related to tumor cells' growth arrest and apoptotic death induced by interacting with DNA and topoisomerase II [46]. In the early 1970s, DNR and DOX were marketed and became the prototypes of the ANTs class [47]. However, the clinical application of DNR and DOX was soon limited by multidrug resistance and severe cardiotoxicity, prompting the discovery of novel analogs [48]. In the past five decades, scientists produced thousands of DNR and DOX derivatives and attempted to discover novel ANTs with superior activity and lower toxicity. Most modifications have been focused on the sugar moiety, such as epirubicin, idarubicin, and pirarubicin [49]. Only a few analogs have been generated by altering the daunosamine, such as mitoxantrone. More recently, researchers reported that ACT requires the combination of two cellular activities, DNA damage and chromatin damage, and proposed the possibility of detoxification with a mini chemical modification to remove the DNA-damaging activity of ANTs [50]. Besides structural modification, efforts have been made to reduce toxicity by enhancing the specific targeting with the change of dosage forms, such as the liposome-encapsulated DOX [51]. Despite efforts to develop “the better ANTs,” ANTs remain the leading cause of chemotherapy-induced cardiotoxicity today [52].

### 4.2. General Trends in ACT Research

Based on the publications relating to ACT between 2002 and 2021 from the WoSCC database, we carried out a bibliometric analysis to gain a comprehensive overview of the research trends concerning ACT during the past two decades and provide some valuable information for further research on this field.

The present study contained 3504 publications, including 2762 articles and 742 reviews. The increasing growth of annual publications and citations from 2002 to 2021 suggests scholars' persistent interest and efforts in the ACT field. With the largest number of publications and citations, the United States ranked the top in the coauthorship analysis network of countries/regions. It may be consistent with the strong support from the United States Department of Health and Human Services and the National Institutes of Health, the two agencies which provide financial support for more than 50% of those sponsored publications. At the same time, the United States owned the most papers published with multiple countries' cooperation and encompassed the strongest collaboration worldwide in this field.

Charles University in the Czech Republic was the most productive institution, while 7 of the top 10 institutions in ACT study were from the United States. These results demonstrate that the United States may have a virtual influence and play a leading role in the direction of ACT research. However, contributions to the field from authors of other countries/regions should not be ignored. From the coauthorship analysis, Simunek Tomas from Charles University, located in the Czech Republic, was pioneered in the ACT field with the largest number of publications. Lipshultz Steven E. from the State University of New York, located in the United States, owned the highest *H*-index, suggesting his outstanding work in this field. In addition, the Journal of Clinical Oncology may be gained more attention for its comprehensive influence in this research field with the highest IF and a large number of publications during the survey period.

### 4.3. Future Outlook in the ACT Research

Our co-occurrence networks, burst analysis, and cluster analysis of keywords and cocited references implied the current frontiers and future directions in research concerning ACT from multiple perspectives.

The co-occurrence network of keywords illustrates five clusters concerning ACT, including the treatment of ANTs, mechanism, clinical studies, evaluated indexes, and detection (Figure 8(a)). The burst analysis of keywords showed that terms of left ventricular ejection fraction and global longitudinal strain might be considered essential topics in this field (Figure 8(b)). It indicates the focus on noninvasive and sensitive indexes in the early detection of functional change induced by ANTs. The thematic map suggested that pharmacogenomics may be a promising research direction for ACT therapy and pharmaceutical exploitation (Figure 8(c)). The most frequent cocited references also implied the attention to the molecular mechanism and early detection of cardiotoxicity induced by ANTs from researchers (Table 3 and Figure 9(a)). Besides, the thematic map showed that cardiooncology, a new-developed crossdiscipline of oncology and cardiology, may be a further frontier in the ACT field (Figure 9(b)). In general, future frontiers included in this field are as follows:

#### 4.3.1. Early Detection of ACT

There is increasing emphasis on the early use of biomarkers to detect cardiotoxicity before it becomes irreversible [53]. The biomarkers in most ACT investigations have focused on cardiac troponins and natriuretic peptides, two types of well-established biomarkers for cardiac injury [54, 55]. Cardiac troponins, which mainly include cardiac troponins I (cTnI), cardiac troponins T (cTnT), and high sensitivity troponin I (hs-TnI), are blood biomarkers identified to detect cardiac damage. They are a kind of medium-sized protein which could significantly increase within 2 or 3 hours after cardiac injury [56]. Among those indexes, hs-TnI represents the sensitive change of abnormal myocardial status during ANT treatments [57]. And compared to cTnT, a persistent elevation of cTnI is associated with a greater degree of left ventricular dysfunction and a higher incidence of cardiac events [58]. Natriuretic peptides mainly include BNP and N-terminal pro-B-type natriuretic peptide (NT-proBNP). BNP is a 32-amino acid protein with natriuretic, diuretic and vasodilator properties [59]. NT-proBNP is an amino-terminal fragment of BNP that shows more stability in detection. Detection of BNP and NT-proBNP after 24 h upon ANT intake can effectively evaluate early cardiotoxicity [60]. However, according to the strict requirement of detected time points, levels of cardiac troponins and natriuretic peptides are usually quite variable in clinical practice, which implies debated reliability with those biomarkers in the accurate evaluation of ACT.

In order to supplement the deficiency of blood biomarkers, echocardiography has been employed in the early detection of cardiotoxicity. The LVEF with a drop of 10% from the baseline to an absolute value of <50% is commonly used in identifying ACT [61, 62]. However, the precise evaluation of LVEF may depend on operator experience and may not be sufficiently sensitive for subclinical myocardial dysfunction [63]. Subsequently, the GLS, an indicator more sensitive than LVEF, has emerged for assessing subclinical myocardial dysfunction. A reduction in GLS of >15% from baseline is generally considered an early sign of heart failure. Recent studies that applied GLS to guide clinical decision-making have reduced the incidence of cancer therapy-related cardiac dysfunction [64]. Accordingly, GLS might be a preferred index for early detection of cardiotoxicity during ANT therapy.

Although there are constant efforts to detect early cardiotoxicity, the perfect biomarkers with noninvasive and economic still have not been found. Future research tends to look for the more sensitive, more stabilized, noninvasive, and affordable diagnostic indicators in the early detection of ACT.

#### 4.3.2. The Molecular Mechanism of ACT

ACT is a significant risk factor limiting ANTs' clinical application, but its exact biological mechanisms are not entirely understood. The reactive oxygen species- (ROS-) driven hypothesis has long dominated ACT research. On the one hand, ANTs can directly promote ROS production and exhaust cardiomyocytes' antioxidant capacity. On the other hand, ANTs could form Fe^3+^-ANT complexes in the presence of iron and then catalyze Fenton's reaction, which would promote H_2_O_2_ converted to ROS. Ultimately, excessive ROS induced by ANTs contributes to cardiomyocyte death [65]. However, several trials found that antioxidants are ineffective for the ACT, indicating a more complex ACT mechanism, which may involve mitochondrial dysfunction and DNA damage [66, 67].

ANTs are proved the potent mitochondrial toxins [68]. Research reported that ANTs could affect the mitochondrial oxidative phosphorylation system by directly interfering with mitochondrial structures, which could induce nuclear-mediated effects of drugs and alter gene expression, resulting in alter of autophagy/mitophagy fluxes and acceleration of cellular death [69]. Meanwhile, ANTs could also induce heart injury by DNA damage. Research suggested that ACT is mediated by Top2*β*, a classical cellular target of DOX, in cardiomyocytes [70]. And the formation of the Top2*β*-DOX-DNA ternary complex would induce DNA double-strand breaks, ultimately resulting in cardiomyocyte death [71].

In addition, programmed cell death concerning ACT also obtains many concerns from researchers. The literature revealed that DOX-induced cardiotoxicity is closely related to autophagy regulation [72]. ANTs could suppress lysosomal proteolysis resulting in autophagosome and autolysosome accumulation, promoting cell death [73]. Apoptosis and pyroptosis-mediated loss of cardiomyocytes also play an essential role in cardiotoxicity. Studies also reported that pyroptosis is more critical than apoptosis in the ACT [74]. Recently, the mechanism of the relationship between ferroptosis, a programmed iron-dependent cell death, and ACT has been explored. Specifically, ANTs trigger ferroptosis via activating nuclear factor erythroid 2-related factor 2 (nrf-2) and upregulating heme oxygenase 1 (hmox1), resulting in the release and accumulation of free iron, which induces ACT [75]. And Ferrostatin-1 (Fer-1), a ferroptosis inhibitor, could significantly improve ACT [76].

In summary, ANT treatment would result in excessive production of ROS, mitochondrial dysfunction, and DNA damage, triggering various cell death pathways and eventually inducing ACT. Although their molecular mechanism has been extensively studied, the optimal therapeutic target remains to be further elucidated.

#### 4.3.3. The Preventive and Treatment for ACT

Clinically, the strategies against ACT usually include decreasing cumulative dose, using liposomal formulations, and employing cardioprotective medications [77].

Due to the dose-dependent cardiotoxicity of ANTs, the cumulative clinical dose is generally limited to 400 mg/m^2^ of DOX and 600 mg/m^2^ of epirubicin [78]. However, this approach only effectively reduces short-term cardiotoxicity but not long-term cardiotoxicity. Thus, scientists turned their eyes to the drug dosage form. Luckily, the liposome-encapsulated ANTs, a low-toxic dosage form of ANTs, have been invented [79]. And liposomal DOX is currently approved by the Food & Drug Administration (FDA) [80]. More recently, researchers combined the Se@SiO_2_ nanocomposite and DOX, from which they discovered the promising effect of Se@SiO_2_ against ACT [81, 82]. Besides, clinicians found that the slower continuous infusion dosing of ANTs could reduce the risk of cardiotoxicity compared with the rapid bolus dosing [83].

So far, dexrazoxane is the only agent approved by the FDA to prevent ACT. However, the risk of secondary malignancies limits dexrazoxane application [84]. Aside from dexrazoxane, there are also many promising prophylactics against ACT in clinical practice. For instance, statins have been proposed as an option for the primary prevention of ACT [85]. The role of angiotensin-converting enzyme inhibitors in secondary prevention is also well established for their well-proven effect on LVEF recovery [86].

In recent years, the emerging view is to repurpose drugs for metabolic diseases in cardiotoxicity treatment [87]. In this scenario, empagliflozin and metformin, two anti-diabetic drugs, have been proven to simultaneously reduce blood glucose levels and rescue heart injury [88]. In addition, promising effects of natural therapeutics and bioactive compounds from herbals in treating disease treatment and improving physical function are also in the spotlight [89–91]. Several studies have confirmed the efficacy of herbal extracts and traditional Chinese medicine injection in preventing and treating ACT, such as saffron extract, Shenfu injection, and Shenmai injection [92–94]. And further studies have been performed to explore the mechanism of active ingredients from herbal extracts against the ACT. For example, cryptotanshinone could treat ACT via the Akt-GSK-3*β*-mPTP pathway [95]. Dihydrotanshinone I could be applied as a potential agent for ACT treatment via the mTOR-TFEB-NF-*κ*B signal pathway [96]. Accordingly, more possibilities and inspiration for anti-ACT could be excavated from natural products.

#### 4.3.4. Cardiooncology and ACT

Cardiooncology is a rapidly growing field in cardiology that focuses on the surveillance, prevention, and management of cardiovascular toxicities and complications caused by anticancer therapies [97, 98]. In 2000, the first cardiooncology unit was established at the MD Anderson Cancer Center in the United States [99]. Cardiooncology research mainly includes cardiotoxicity of anticancer therapy, cardiovascular complications of tumors, risk factors of cardiovascular diseases and tumors, and cardiac tumors. With the development of anticancer agents, the survival of oncology patients has been prolonged. However, their risk of cardiovascular disease was an unexpected increase. ANTs are one of the proven chemotherapeutic drugs with cardiac toxicity [100].

Cardiooncology emphasizes risk stratification and monitoring throughout the ACT treatment by understanding its molecular mechanism. At present, the crosstalk between cardiac and cancer cells has been gaining attention. Studies reported the intersections between cardiac metabolism and cancer biology, and the potential role for carnitine, citric acid, and aconitic acid in the ACT has been highlighted [101]. Besides, cardiooncology studied the possible pathways of ACT with the high-throughput technology and screened out the nuclear enriched abundant transcript 1/let-7f-2-3p/exportin-1 signaling axis [102, 103].

Overall, cardiooncology is at the forefront of an evolving field of medical sciences. It promoted our knowledge of the pathophysiological mechanisms of ACT, which is crucial for evidence-based management strategies in clinical practice [104]. In the near future, cardiooncology should facilitate the translation of research evidence into clinical practice.

#### 4.3.5. Pharmacogenomics and ACT

Pharmacogenomics is an individualized approach to determining inherited differences in drug disposition and treatment response [105]. It aimed to identify markers predictive of adverse effects, enhance drug efficacy, and reduce toxicity [106]. Emerging evidence established significant polygenic contributions that predispose to ACT. And pharmacogenomics could be used to identify those at higher risk of complications [107]. For instance, literature reported that a missense variant rs2229774 in the retinoic acid receptor-*γ* (RARG) gene is associated with increased susceptibility to ACT. At the same time, RARG agonist treatment has the potential to further protect patients with or without the rs2229774 variant from cardiotoxicity [108, 109]. POLRMT, a gene that encodes a mitochondrial DNA-directed RNA polymerase, was discovered as a novel susceptibility gene for the act of breast cancer patients in a genome-wide association study [110]. Similarly, the multiple genetic variants in the solute carrier family 28 member 3 (SLC28A3) could distinguish child patients with high or low risk for the ACT, and researchers identified the solute carrier (SLC) competitive inhibitor can effectively against ACT [111, 112]. In addition, recent research indicated that the glutathione S-transferase [GST] *μ*1 (GSTM1) appears to be an important gene in predisposition to ACT in survivors of childhood cancer [113]. To date, multiple genes and intergenic variants have now emerged as risk loci for ACT, including the solute carrier family 22 member 7 (SLC22A7), the ATP binding cassette subfamily C member 1 (ABCC1), the carbonyl reductase 3 (CBR3), the neutrophil cytosolic factor 4 (NCF4), and transient receptor potential cation channel subunit 6 (TRPC6) [114, 115]. Recently, a gene regulatory network has been constructed and further screened several key ones among identified genes, including the ryanodine receptor 2 (RYR2), the tumor necrosis factor receptor superfamily member 12A (TNFRSF12A), and the sodium voltage-gated channel beta subunit 3 (SCN3B) [116].

The development of pharmacogenomics facilitated the discovery of a novel molecular mechanism involved in the ACT and improved our ability to predict patients who are at risk. However, the known genetic risk factors do not fully explain the interindividual variability. They can only predict which patients are more likely to develop this severe toxicity. The larger-scale studies are needed for further identification. Pharmacogenomics research has an apparent implication for improving ACT treatment outcomes, representing a promising research area. Pharmacogenomics will hopefully result in personalized therapies that will help vulnerable patients to be safely cured of cancer.

### 4.4. Limitations

The present study provided a comprehensive overview of the global status and research trends in the ACT field during the past 20 years using bibliometric analysis for the first time. Compared to traditional literature reviews, the bibliometric study is relatively more objective. Nevertheless, several limitations in the bibliometric research should also be considered. Firstly, although the WoSCC is the most commonly used database for bibliometric analysis, there are many other literature databases, such as PubMed and Embase. With the development of bibliometric software's function that simultaneously analyzes literature from distinct databases, more publications from different databases should be included. Secondly, some recently published important papers might not gain enough citations, leading to the omission of important information. Thus, it is necessary to make bibliometric analyses at intervals to look back at some important themes in a research field. Thirdly, there are some differences between bibliometric analysis results and real-world research conditions. For instance, due to the complex and active cooperation, the actual contribution of authors or institutions could not be totally identified with bibliometric applications.

## 5. Conclusions

The present study is the first bibliometric analysis of ACT. It provides a comprehensive and detailed overview of development trends and research frontiers of the ACT field from published academic literature. According to analysis, the United States significantly contributed to the ACT research from multiple aspects, including publication, citations, institutions, funding agencies, and collaboration worldwide. Simunek Tomas from Charles University and Lipshultz Steven E. from the State University of New York were the two outstanding scientists who significantly impacted this field. The Journal of Clinical Oncology was significant in the area. The burst and cluster analysis of keywords and cocited references indicated that the future research frontiers of ACT might include early detection, pharmacogenomics, molecular mechanism, and cardiooncology. The topics regarding ACT deserve continued follow-up by researchers. This study could provide a valuable reference for researchers and practitioners of the field in future research directions.

## Figures and Tables

**Table 1:** The top 10 productive countries/regions in the ACT field.

Rank	Countries/regions	Publications	Rate (*N*/3504) %	Citations
1	The United States (North America)	1154	32.93	55528
2	Italy (Europe)	442	12.61	23065
3	China (Asia)	343	9.79	6960
4	Canada (North America)	271	7.73	16395
5	Germany (Europe)	196	5.59	9672
6	England (Europe)	192	5.48	10594
7	Netherlands (Europe)	167	4.77	10332
8	Japan (Asia)	139	3.97	2383
9	France (Europe)	136	3.88	9107
10	Australia (Oceania)	118	3.37	10214

**Table 2:** The top 10 productive institutions in the ACT field.

Rank	Institutions	Countries	Publications	Rate (*N*/3504) %
1	Charles University	The Czech Republic	74	2.11%
2	The University of Texas MD Anderson Cancer Center	The United States	73	2.08%
3	Memorial Sloan Kettering Cancer Center	The United States	63	1.80%
4	University of Toronto	Canada	55	1.57%
5	University of Pennsylvania	The United States	53	1.51%
6	Mayo Clinic	The United States	52	1.48%
7	University of Naples Federico II	Italy	46	1.31%
8	Stanford University	The United States	46	1.31%
9	St. Jude Children's Research Hospital	The United States	45	1.28%
10	Harvard University	The United States	43	1.23%

**Table 3:** The top 10 cocited references in the ACT field.

Citations	Bursts	Title	Authors	Year	Journal
365	40.64	Identification of the molecular basis of doxorubicin-induced cardiotoxicity	Zhang et al.	2012	Nat Med
320	77.47	Early detection of anthracycline cardiotoxicity and improvement with heart failure therapy	Cardinale et al.	2015	Circulation
275	84.43	Anthracyclines: molecular advances and pharmacologic developments in antitumor activity and cardiotoxicity	Minotti et al.	2004	Pharmacol Rev
258	23.24	Anthracycline-induced cardiomyopathy: clinical relevance and response to pharmacologic therapy	Cardinale et al.	2010	J Am Coll Cardiol
243	76.73	2016 ESC Position Paper on cancer treatments and cardiovascular toxicity developed under the auspices of the ESC Committee for Practice Guidelines: the task force for cancer treatments and cardiovascular toxicity of the European Society of Cardiology (ESC)	Zamorano et al.	2016	Eur Heart J
234	44.4	Expert consensus for multimodality imaging evaluation of adult patients during and after cancer therapy: a report from the American Society of Echocardiography and the European Association of Cardiovascular Imaging	Plana et al.	2014	J Am Soc Echocardiogr
220	21.91	Assessment of echocardiography and biomarkers for the extended prediction of cardiotoxicity in patients treated with anthracyclines, taxanes, and trastuzumab	Sawaya et al.	2012	Circ Cardiovasc Imaging
190	32.5	Use of myocardial strain imaging by echocardiography for the early detection of cardiotoxicity in patients during and after cancer chemotherapy	Thavendiranathan et al.	2014	J Am Coll Cardiol
184	29.15	Anthracycline-induced cardiotoxicity: overview of studies examining the roles of oxidative stress and free cellular iron	Simunek et al.	2009	Pharmacol Rep
182	12.59	Early detection and prediction of cardiotoxicity in chemotherapy-treated patients	Sawaya et al.	2011	Am J Cardiol

**Table 4:** The top 10 largest clusters of cocited references in the ACT field.

Cluster ID	Size	Silhouette	Mean year	Label
0	38	0.971	2010	Strain
1	30	0.904	1997	Natriuretic peptides
2	29	0.901	1999	Metastatic breast cancer
3	29	0.985	2013	Cardiooncology
4	28	0.977	2009	Chemotherapy
5	25	0.965	2013	Doxorubicin
6	25	0.942	2000	Adverse effects
7	24	0.888	2001	Apoptosis
8	24	0.989	2005	Trastuzumab
9	23	0.905	1998	Dexrazoxane

## Data Availability

The datasets generated for this study are available upon request to the corresponding author.
